# Scope of public health measures in ensuring road safety

**DOI:** 10.5249/jivr.v6i2.542

**Published:** 2014-07

**Authors:** Saurabh RamBihariLal Shrivastava, Prateek Saurabh Shrivastava, Jegadeesh Ramasamy

**Affiliations:** ^*a*^Department of Community Medicine, Shri Sathya Sai Medical College & Research Institute, Kancheepuram, India.

Globally, 1.24 million people succumb to road traffic accidents (RTA) every year, 92% of which have been reported from low and middle-income countries.^1^ It raises further concerns as almost 60% of the victims are from 15-44 years age group.^1^ Realizing the public health concern, the current decade 2011-2020 has been declared as the Decade of Action for road safety, with a goal of first stabilizing and then decreasing the anticipated magnitude of RTA associated mortality by augmenting the global efforts at national and international level.^2^

A heterogeneous group of socioeconomic factors such as male gender;^3^ young age;^3^ poor education;^3,4^ low income;^4^ risk taking behavior;^4^ alcohol consumption;^1,5^ psychoactive substances;^5^ non-use of personal protective equipments;^1,4^ non-compliance with the traffic safety rules and speed limits;^1,4^ riding motorcycles or heavy motor vehicles;^3,4^ duration between the accident and getting the driving license;^4^ passengers occupying the front seats;^4^ and delay in provision of prompt medical care to the victims of road traffic accidents;^1,6^ have been identified as the potential risk factors in the causation or amplification of the aftermaths of the accidents.

Since 2007, 88 countries have shown a significant reduction in the mortality associated with RTA, providing a ray of hope that sufficient political commitment can result in remarkable progress.^1^ The vision is to develop a national strategy by setting realistic targets for the benefit of vulnerable road users (viz. pedestrians, cyclists, and riders of motorized two-wheelers), vehicles and the road environment in an integrated manner through implementation of a wide range of interventions.^3,4^ Primary step is to develop a network for data collection to identify the causative factors and the accurate magnitude of RTA so that rational policy can be planned for achieving the best possible allocation of limited resources, especially in developing countries.^1,4^ Other measures such as public awareness campaigns for educating the masses through the use of mass media about road safety rules and regulations / need of wearing personal protective equipments / penalties associated;^4^ enabling involvement of multiple sectors in National road safety efforts;^2^ strict enforcement of legislations advocating the use of seat-belts, helmets and child restraint;^1,2^ setting and enforcing blood alcohol concentration limits for drivers;^4,5^ designing safer roads;^2^ engineering measures to enhance the vehicle safety standards;^2,3^ advocating safer modes of public transport;^4^ promoting effective land-use, urban planning, no traffic zone;^4^ establishing prompt and good quality post-crash response;^4,6^ and establishing monitoring and evaluation system to assess the outcome of implemented measures;^2,4^ can be strategically implemented to counter the burden of RTA.

To conclude global implementation of preventive and control measures supported by political commitment can save countless number of lives and concurrently reduce the burden on the health care system. 

## References

[1] World Health Organization. 10 facts on global road safety. 2013, http://www.who.int/features/factfiles/roadsafety/en/index.html,accessed 5 May 2013.

[2] World Health Organization. Global Plan for the Decade of Action for Road Safety 2011-2020. 2010, http://www.who.int/roadsafety/decade_of_action/plan/en/,accessed 2o June 2012.

[3] Kanchan T, Kulkarni V, Bakkannavar SM, Kumar N, Unnikrishnan B (2012). Analysis of fatal road traffic accidents in a coastal township of South India. J Forensic Leg Med.

[4] World Health Organization. Global status report on road safety, 2013- Supporting a decade of action. Geneva : WHO Press,2013.

[5] Bogstrand ST, Gjerde H, Normann PT, Rossow I, Ekeberg O (2012). Alcohol, psychoactive substances and non-fatal road traffic accidents--a case-control study. BMC Public Health.

[6] Sanchez-Mangas R, García-Ferrrer A, de Juan A, Arroyo AM (2010). The probability of death in road traffic accidents. How important is a quick medical response?. Accid Anal Prev.

